# Stable Resistance to Potato Virus Y and Potato Leafroll Virus in Transgenic Potato Plants cv. Kennebec Expressing Viral Genes Under Greenhouse and Field Conditions

**DOI:** 10.3390/plants15030355

**Published:** 2026-01-23

**Authors:** María Pilar Barrios Barón, Natalia Inés Almasia, Vanesa Nahirñak, Diego Zavallo, Deimer Daniel Rodriguez Diaz, Sebastián Asurmendi, Federico Fuligna, Horacio Esteban Hopp, Ana Julia Distéfano, Cecilia Vazquez Rovere

**Affiliations:** 1 Instituto de Agrobiotecnología y Biología Molecular (IABIMO) UEDD INTA-CONICET, Instituto de Biotecnología, Centro de Investigaciones de Ciencias Veterinarias y Agronómicas INTA, Hurlingham B1686, Argentina; mpilarbb@gmail.com (M.P.B.B.); nahirnak.vanesa@inta.gob.ar (V.N.); dzavallo@gmail.com (D.Z.); rodriguezdiaz.daniel@inta.gob.ar (D.D.R.D.); asurmendi.sebastian@inta.gob.ar (S.A.); or ehopp@fbmc.fcen.uba.ar (H.E.H.); distefano.ana@inta.gob.ar (A.J.D.); vazquez.cecilia@inta.gob.ar (C.V.R.); 2 EEA-INTA AER La Consulta, Ex Ruta 40 Km 96, La Consulta, San Carlos 5567, Argentina; fuligna.federico@inta.gob.ar; 3 Facultad de Ciencias Exactas y Naturales, Universidad de Buenos Aires, Intendente Güiraldes 2160, Ciudad Autónoma de Buenos Aires C1428, Argentina

**Keywords:** potato virus Y, potato leafroll virus, potatoes, field trials, mixed infections, agronomic performance

## Abstract

Potato virus Y (PVY) and potato leafroll virus (PLRV) are the most damaging viruses for potato production worldwide. Mixed infections not only result in additive detrimental effects on plant growth and tuber yield but also complicate the development of durable and broad-spectrum viral resistance. Heterologous protection against PVY can be achieved through the expression of the coat protein (CP) of lettuce mosaic virus (LMV) (CPLMV), conferring resistance via a capsid protein-mediated mechanism. On the other hand, we have previously demonstrated that transgenic lines expressing the PLRV ORF2 (RepPLRV) exhibit resistance to different PLRV isolates. In this study, potato transgenic lines of cv. Kennebec expressing CPLMV and RepPLRV were developed to confer dual virus resistance. Transgenic and non-transgenic control plants were molecularly and phenotypically characterized in greenhouse and field conditions. Across multiple growing seasons, two selected transgenic lines consistently displayed robust resistance to both major viruses, without exhibiting yield penalties or noticeable phenotypic alterations. These results constitute a significant advancement, demonstrating that dual resistance to PVY and PLRV can be achieved while preserving the original agronomic performance of the cultivar. This breakthrough not only contributes to long-term crop productivity but also provides a more sustainable strategy for managing viral diseases in potato production.

## 1. Introduction

The potato (*Solanum tuberosum* L.) is an herbaceous plant belonging to the *Solanaceae* family and is considered one of the world’s most important food crops. It serves as a major staple food that plays a substantial role in feeding the world’s growing population [1]. It is currently cultivated in more than 100 countries on an estimated 16.8 million hectares of farmland, and 383 million tonnes of potatoes were produced globally in 2023 (The Food and Agriculture Organization’s database, updated in late December 2024). Argentina produces approximately 2.8 million tonnes, allocating approximately 75–80 thousand hectares to potato cultivation. Potatoes are the most widely consumed vegetable in the country, and their consumption has shown a positive trend over the years [2]. The main variety of potato grown and marketed in Argentina for fresh consumption is Spunta, while the Kennebec variety is important in terms of industrial production, as it contains between 18 and 19% dry matter and is suitable for making sticks and puree [2]. Spunta and Kennebec potato varieties differ in their yield, with Kennebec generally showing higher overall yield and better performance under stress conditions, whereas Spunta is more responsive to external factors like biostimulants, which can increase tuber numbers [3].

Virus infections are a major threat to potato production since they can significantly decrease not only yield but also tuber quality. To date, approximately fifty viruses and one viroid have been reported to naturally infect *S. tuberosum* [4]. Among them, *Potato virus* Y (PVY, genus *Potyvirus*, family *Potyviridae*) and *Potato leafroll virus* (PLRV, genus *Polerovirus*, family *Solemoviridae*) are the most important and damaging potato viruses in the world [4]. Both are transmitted by aphids (in a non-persistent or persistent manner, respectively) and are prevalent in most potato-growing areas in the world [5,6,7]. PVY can reduce total yield and marketable yield by 49% and 65%, respectively [8]. Similarly, PLRV-infected seed tubers were reported to result in losses in total yield of 60% and in marketable tuber yield of 88% [8]. Of note, studies from China, the world’s largest potato-producing country, showed that co-infection of PVY and PLRV causes much greater yield loss than single PVY or PLRV infections [7]. Moreover, there are reports of mixed PVY and PLRV infections in potatoes worldwide [9]. Both viruses coexist stably in nature, causing additive or synergistic effects on crop growth and productivity, thereby highlighting the importance of using virus-free seed potatoes or resistant varieties to reduce the impact of such infections [10].

Natural complete resistance to PVY and PLRV has not been incorporated, to date, in actual commercial potato cultivars [6,11,12,13,14]. Despite the paucity of sources of complete resistance [15], potato breeders have introduced different types and sources of resistance to PVY and PLRV, originally identified in wild *Solanum* germplasm, into some potato cultivars [16,17,18,19,20]. Nevertheless, introgression is a laborious process, and, to date, many commercial cultivars remain susceptible. Consequently, at present, potato producers mostly manage diseases caused by PVY and PLRV using certified seeds whose main constraint is the high cost, and/or by insecticide applications with the entailing economic and environmental concerns [21,22,23]. Genetic engineering of potatoes to confer virus resistance offers a more sustainable approach with reduced environmental impact. It also overcomes key challenges in conventional breeding, such as the complexity of tetraploid potato genetics and the need for large populations, whilst shortening times and maintaining the elite cultivar genetic background except for the introduced trait [19,24].

Capsid protein (CP)-mediated resistance was one of the first transgenic approaches shown to confer virus resistance (or tolerance) in plants [25]. High levels of resistance have been reported in transgenic plants expressing the CP of several RNA viruses, including tobacco mosaic virus (TMV), potato virus X (PVX), cucumber mosaic virus (CMV), and tobacco rattle virus (TRV), suggesting that CP-mediated resistance may interfere with viral disassembly during infection [26]. Heterologous protection against several strains of PVY, as well as other potyviruses, can be reached through the expression of the CP of the lettuce mosaic virus (LMV) (CPLMV) in transgenic tobacco plants [27]. Although this protection was associated with detectable levels of CPLMV accumulation, there was no clear correlation between the level of the protein expression and the degree of protection [28]. Furthermore, Hassairi et al. [29] reported similar results in greenhouse evaluations of two potato cultivars transformed with the CPLMV coding sequence. Another strategy for achieving viral resistance involves the RNA silencing mechanism. We have previously developed transgenic lines of the potato cultivar Kennebec expressing ORF2 from an Argentinian PLRV isolate (hereafter referred to as RepPLRV). These lines exhibited resistance to different PLRV isolates, as confirmed by both grafting assays and field trials. Furthermore, the protection mechanism was suggested to be mediated by RNA silencing [30]. In addition to the detrimental additive effects on crop growth and productivity of mixed infections, the simultaneous presence of multiple viruses poses a significant challenge to the efficacy of RNA silencing as a strategy for viral resistance [31]. This is because certain viruses encode potent viral suppressors of RNA silencing that compromise the efficiency of this mechanism. Indeed, PVY encodes the Hc-Pro protein, a well-known and strong viral suppressor of RNA. Thus, under natural field conditions, in cases of a mixed infection, PVY could potentially overcome or interfere with engineered resistance against PLRV [30,32].

In this context, and with the aim of obtaining transgenic plants stably resistant to PVY and PLRV, our group developed transgenic cv. Kennebec plants expressing the coding sequence for the CPLMV protein as well as the RepPLRV. These transgenic cv. Kennebec potato lines were molecularly and phenotypically characterized, and their resistance to infections caused by both viruses was evaluated. This study represents a significant achievement, demonstrating the successful development of commercially valuable potato lines resistant to both PVY and PLRV, while preserving the agronomic performance of the original cultivar under both greenhouse and field conditions.

## 2. Results

### 2.1. The Generation of Transgenic Plants Engineered to Express the Coat Protein of Lettuce Mosaic Virus and the ORF2 Sequence of Potato Leafroll Virus

**Figure 1:**
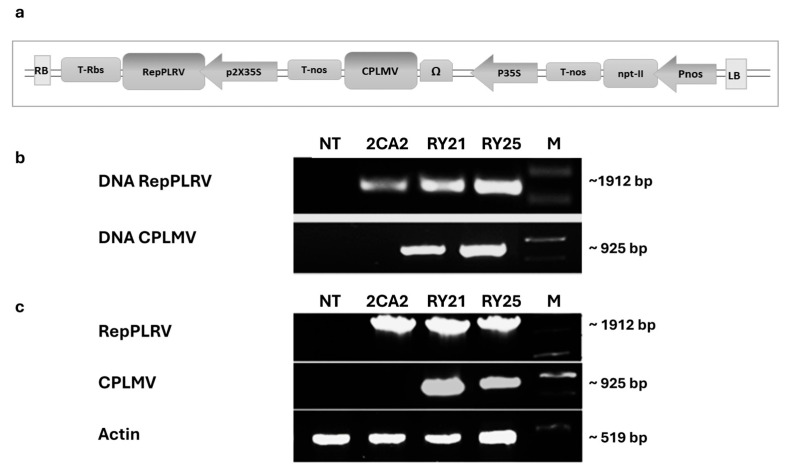
Development of transgenic plants engineered to express the coat protein of lettuce mosaic virus (CPLMV) and the ORF2 sequence of potato leafroll virus (RepPLRV). (**a**) Schematic representation of the expression cassette in the binary vector pPZP-Kan-CPLMV-RepPLRV used for the transformation of *S. tuberosum* cv. Kennebec plants. Diagrams are not to scale. P35S and 2 × 35S: single and double CaMV-35S promoters, respectively; Pnos: nopaline synthase promoter; Ω: synthetic 65 bp leader sequence from tobacco mosaic virus; nptII: neomycin phosphotransferase II; RepPLRV: ORF2 of PLRV; CPLMV: LMV capsid protein; Tnos: nopaline synthase terminator; T-Rbs: rubisco terminator; RB and LB: right- and left-border sequences of the T-DNA region, respectively. (**b**) PCR amplification of fragments corresponding to the complete RepPLRV sequence and to the complete ORF encoding CPLMV. (**c**) RT-PCR analysis performed using total RNA from the transgenic lines. Amplified PCR products are indicated on the left, and product sizes are shown on the right. NT: Non-transgenic control potato cv. Kennebec; 2CA2: single transgenic line carrying only the cassette encoding RepPLRV; RY21 and RY25: transgenic lines expressing both RepPLRV and CPLMV. M: 1 kb DNA molecular weight marker.

With the aim of obtaining transgenic potato plants simultaneously resistant to PVY and PLRV, we developed a unique construct containing cassettes that enable the expression of the coat protein gene of the lettuce mosaic virus (CPLMV) and the ORF2 of potato leafroll virus (RepPLRV), along with a cassette for the expression of the gene *nptII*, (pPZP-Kan-CPLMV-RepPLRV binary vector, Figure 1a), based on a PZP200 vector backbone [33]. Using the *Agrobacterium tumefaciens*-based method, we successfully generated 27 transgenic lines of cv. Kennebec, hereafter referred to as RY, carrying both CPLMV and RepPLRV transgenes. Two promising RY lines, designated as RY21 and RY25, were selected to conduct further analysis. This selection was based on superior transcript levels of CPLMV and stable PLRV expression as determined by RT-PCR assays [34]; also, preliminary greenhouse screenings allowed us to exclude off-type lines, ensuring morphological uniformity. Subsequently, agronomic traits, including vegetative growth, reproductive yield, general plant vigor, and overall phytosanitary status, were evaluated under field conditions over two consecutive seasons (2005–2007) in Malargüe, Mendoza, Argentina [34]. In addition, the 2CA2 line, a PLRV-resistant Kennebec transgenic line expressing only the RepPLRV, transformed with pVH-ATG-rep and described in Vazquez Rovere et al. [30], was also included as a control in the experiments. First, the integrity of the transgenes in the selected potato lines was assessed using PCR techniques. As shown in Figure 1b, an approximately 1912 bp expected fragment corresponding to the complete RepPLRV was successfully amplified in the RY21, RY25, and 2CA2 lines, while an approximately 925 bp fragment corresponding to the complete CPLMV was detected exclusively in the two RY candidate lines. As expected, no amplification was observed in non-transgenic control plants (NT).

Next, reverse transcriptase PCR (RT-PCR) was performed to verify the transcription of RepPLRV and CPLMV. As shown in Figure 1c, a ~1912 bp fragment corresponding to RepPLRV was amplified from the cDNA of RY and 2CA2 transgenic lines, while a ~925 bp CPLMV fragment was detected exclusively in the cDNA of RY lines. As expected, no transgene amplification was observed in NT control plants, whereas the housekeeping control transcript was properly detected in all samples. Since the actin gene contains an intron in its sequence, the presence of only a single product corresponding to the spliced messenger confirmed the absence of genomic DNA contamination in the synthesized cDNA samples (Figure 1c).

### 2.2. Assessment of Key Molecules Involved in the Resistance Mechanism of RY Transgenic Lines

**Figure 2:**
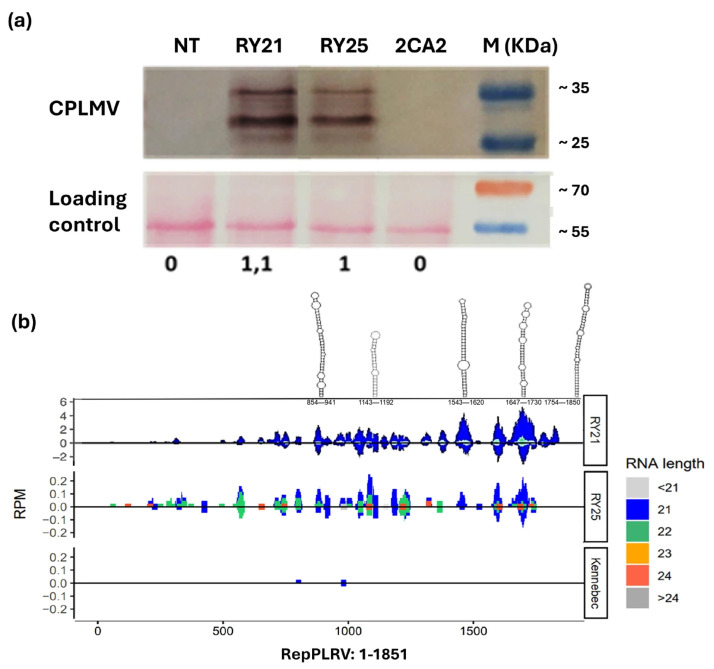
The characterization of the molecules related to the resistance mechanism of the RY transgenic lines. (**a**) Detection of CPLMV in transgenic plants. Western blot analysis was performed using total protein extracts from the transgenic lines. Proteins from non-transformed plants were included as controls. M: protein molecular weight marker; kDa: kilodalton. Bands corresponding to the target protein were detected using a commercial polyclonal antibody, and the estimated marker product sizes are indicated on the right. Ponceau S staining of the membrane was used as the loading control. The lower panel shows protein expression in arbitrary units relative to the amount of loaded protein. (**b**) Detection of interfering RNA (siRNA) duplexes induced by RepPLRV silencing. The distribution of viral reads obtained by next-generation sequencing (NGS) and mapped to the RepPLRV sequence is shown. Regions of strong secondary structure within the RepPLRV RNA were modeled and are depicted along the upper border. Graphs are presented at different scales to improve the visualization of the mapped regions. RPM: reads per million. NT: non-transgenic control potato cv. Kennebec; 2CA2: single transgenic line carrying only the cassette encoding RepPLRV; RY21 and RY25: transgenic lines expressing both RepPLRV and CPLMV.

Since the presence of the CPLMV protein has been shown to be essential for PVY protection in transgenic lines [27], its expression was evaluated in total protein extracts from both RY lines using commercial polyclonal antibodies against the LMV capsid protein. As shown in Figure 2a, the CPLMV protein was clearly detected in the RY21 and RY25 samples, as indicated by strong bands at the expected molecular weight (~31 kDa) (Figure 2a, top panel). Additionally, a band of approximately 35 kDa was observed, likely due to post-translational modification of the CPLMV protein. As expected, no CPLMV signal was observed in NT Kennebec and 2CA2 samples.

Subsequently, since RepPLRV has been suggested as a target of post-transcriptional gene silencing [30], we performed small RNA sequencing to identify specific small interfering RNA (siRNA) associated with RepPLRV silencing. As shown in Figure 2b, next-generation sequencing (NGS) revealed numerous 21 to 24 bp siRNAs distributed heterogeneously along the RepPLRV sequence in RY21 and, to a lesser extent, in RY25. Although the number of reads mapping to RepPLRV was higher for RY21 than for RY25 (3000 vs. 130), both lines exhibited siRNA peaks in the same regions. Notably, duplex structures were modeled in areas with the highest read density, corresponding to regions of strong secondary structure within the RepPLRV RNA. As expected, only two reads from the wild-type Kennebec plant mapped to the RepPLRV sequence (Figure 2b), whereas no relevant results were identified for CPLMV (fewer than 30 reads).

### 2.3. Assessment of PVY Viral Resistance of Transgenic Potato Lines Under Greenhouse Conditions

**Figure 3:**
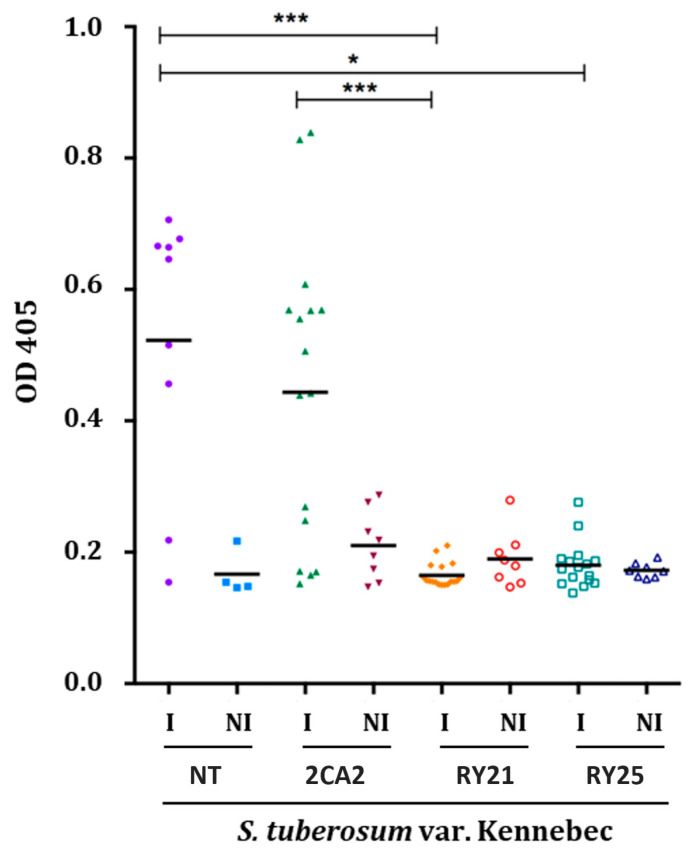
Assessment of selected transgenic potato lines’ resistance to mechanical infection with PVY under greenhouse conditions. Assays were conducted three times and analyzed separately. Since the results were consistent, only data from a representative assay are shown. Treatment groups included non-infected (NI) controls, consisting of NT plants (*N* = 4) and transgenic lines 2CA2, RY21, and RY25 (*N* = 8 per line), and infected (I) groups comprising NT (*N* = 10) and transgenic lines 2CA2, RY21, and RY25 (*N* = 16 per line). Optical density at 405 nm (OD 405) was measured using the ELISA for PVY. NT: non-transgenic control potato cv. Kennebec; 2CA2: transgenic line carrying only the cassette encoding RepPLRV; RY21 and RY25: transgenic lines carrying both RepPLRV and CPLMV. Statistical analyses were performed by two-way ANOVA (GraphPad Prism 8), with statistical significance set at *p* < 0.05. “*” denotes p between 0.01 and 0.05 and “***” between 0.001 and 0.0001.

To assess the resistance of the selected RY transgenic plants under high PVY infection pressure, a mechanical inoculation trial was performed on potato plants using recombinant PVY^NTN^ and necrotrophic PVY^N^ strains collected and previously characterized from the field samples in Mendoza [34]. Inoculation conditions were optimized to achieve an infection rate of approximately 100% in NT plants. To evaluate the resistance of transgenic RY21 and RY25 lines to PVY infection, virus accumulation was measured in systemic leaf samples three weeks post-inoculation using an ELISA (Figure 3). As expected, NT and 2CA2-infected plants exhibited significantly higher OD values, indicating substantial PVY accumulation. In contrast, RY21- and RY25-infected plants displayed significantly lower OD values, comparable to their respective non-infected controls, suggesting effective suppression of viral accumulation. Statistical analysis confirmed these differences, with highly significant reductions in OD values observed for RY21 and RY25 compared to NT. This evaluation was conducted three times, yielding consistent results. These results indicate that the RY21 and RY25 transgenic lines exhibit strong resistance to PVY, even under conditions of high infection pressure induced by mechanical inoculation.

### 2.4. Evaluation of the Growth Parameters of Transgenic Potato Lines Under Greenhouse Conditions

**Figure 4:**
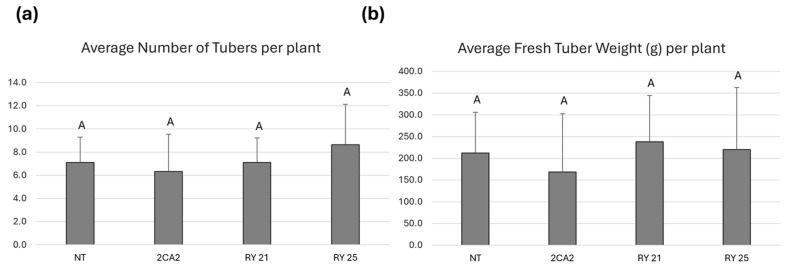
Tuber yield of transgenic lines of *S. tuberosum* cv. Kennebec under greenhouse conditions. A single plant was placed in each pot. Results are based on 25 independent replicates. (**a**) Average number of tubers obtained per plant. (**b**) Average fresh tuber weight per plant (in grams, g). Bars indicate the standard deviation for each line. Statistical analyses were performed using the nonparametric Kruskal–Wallis test with Conover’s post-hoc comparisons, conducted in InfoStat (http://www.infostat.com.ar, accessed on 19 November 2025). Identical letters (e.g., ‘A’) indicate no significant difference (*p* > 0.05) between groups. 2CA2: transgenic line carrying only the cassette encoding RepPLRV; RY21 and RY25: transgenic lines carrying both RepPLRV and CPLMV.

Tuber production was evaluated in transgenic lines RY21 and RY25, as well as in NT and 2CA2 control plants under pathogen-free conditions. The average number of tubers per plant is presented in Figure 4a. No statistically significant differences in tuber numbers were observed among RY21 and RY25 lines compared to the controls. Tuber fresh weight per plant was also measured as an indicator of total yield (Figure 4b), and no statistically significant differences in average fresh weight were observed among the transgenic lines and the NT or 2CA2 controls.

Throughout the greenhouse trials, the RY transgenic lines were phenotypically indistinguishable from the NT control cultivar Kennebec in terms of overall plant morphology, including plant size, leaf shape, and coloration. Post-harvest evaluation of tuber characteristics further revealed no observable differences between transgenic and NT lines with respect to tuber shape, size, flesh color, or skin texture. 

These findings indicate that expression of the RepPLRV and CPLMV transgenes in RY21 and RY25 does not affect tuber number, yield, or overall plant and tuber morphology under the tested greenhouse conditions. In sum, the transgenic lines exhibited growth and developmental parameters comparable to the non-transformed commercial cultivar from which they were derived.

### 2.5. Assessment of Tuber Yield Under Field Conditions

**Figure 5:**
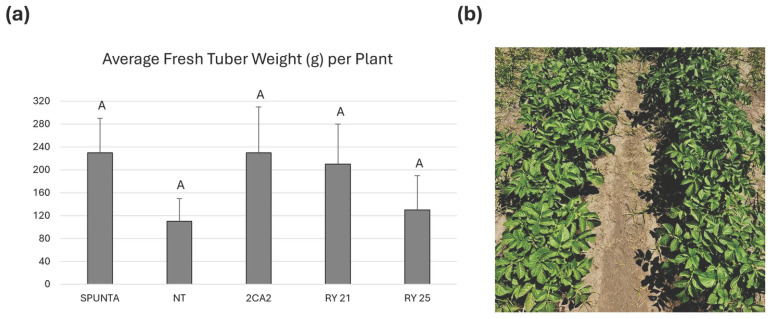
Agronomic performance of transgenic *S. tuberosum* cv. Kennebec lines during the 2015/2016 growing season at the Malargüe experimental field. (**a**) Average fresh tuber weight (g) calculated from the mean value (total tuber weight divided by the total number of plants) for each block. Bars represent the standard deviation of each line, considering each block as a replicate. Statistical analyses were performed using the nonparametric Kruskal–Wallis test with Conover’s post-hoc comparisons, conducted in InfoStat (http://www.infostat.com.ar, accessed on 19 November 2025). Identical letters (e.g., ‘A’) indicate no significant difference (*p* > 0.05) between groups. (**b**) Representative photograph of NT plants (right) and RY25 transgenic plants (left). Spunta, non-transgenic commercial variety; NT, non-transgenic control potato cv. Kennebec; 2CA2, transgenic line carrying only the cassette encoding RepPLRV; RY21 and RY25, transgenic lines carrying both RepPLRV and CPLMV.

To assess the agronomic performance of selected transgenic lines under optimal, virus-free conditions and to compare them with both the parental cultivar Kennebec and the widely grown commercial variety Spunta, field trials were first conducted during the 2016 growing season in Malargüe, Mendoza Province, Argentina, an officially declared virus-free zone. Tubers harvested from greenhouse-grown plants were used. In this field trial, the mean tuber weight per plant was calculated based on the number of plants grown for each line and compared with those of the commercial cultivars Kennebec and Spunta (Figure 5a). The cultivar Spunta exhibited the highest mean tuber fresh weight, averaging 230 g per plant. NT control plants showed a lower average tuber fresh weight (110 g). Notably, transgenic lines 2CA2, RY21, and RY25, with average tuber fresh weights of 230, 210 and 130 g per plant, respectively, showed no significant differences in mean tuber weight compared to either commercial cultivar (NT Kennebec and Spunta) (Figure 5a). In addition, the general phenotypic appearance of the plants was assessed, revealing no noticeable phenotypic differences between the RY transgenic lines and NT controls (Figure 5b).

**Figure 6:**
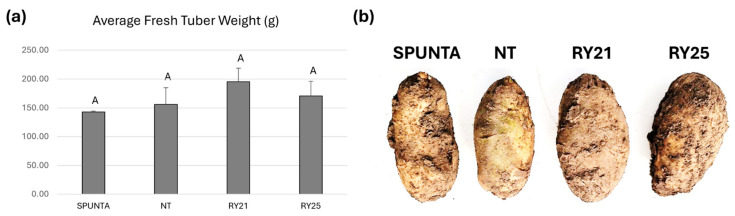
Agronomic performance of transgenic *S. tuberosum* cv. Kennebec lines during three consecutive planting campaigns conducted in Buenos Aires Province, Argentina, between 2022 and 2024. (**a**) Average fresh tuber weight (g) calculated from the mean value (total tuber weight divided by the total number of tubers) for each block. Bars represent the standard deviation of each line, considering each block as a replicate. Statistical analyses were performed using the nonparametric Kruskal–Wallis test with Conover’s post-hoc comparisons, conducted in InfoStat (http://www.infostat.com.ar, accessed on 19 November 2025). Identical letters (e.g., ‘A’) indicate no significant difference (*p* > 0.05) between groups. (**b**) Representative photograph of harvested tubers. Spunta, non-transgenic commercial variety; NT, non-transgenic control potato cv. Kennebec; RY21 and RY25, transgenic lines carrying both RepPLRV and CPLMV.

Moreover, three additional consecutive planting campaigns were conducted in a field in Buenos Aires Province, Argentina, between 2022 and 2024. Tuber yield was evaluated across these field trials, and the average tuber weight produced by the transgenic lines did not differ significantly from that of the NT or Spunta controls (Figure 6a). Post-harvest evaluation of tuber characteristics revealed no noticeable differences between transgenic and non-transgenic lines with respect to tuber shape, size, or skin texture (Figure 6b).

The results obtained under natural field conditions in two distinct regions, Mendoza and Buenos Aires Province, separated by 1080 km, demonstrate that the transgenic lines RY21 and RY25 exhibit agronomic performance comparable to that of the non-transformed Kennebec cultivar from which they were derived. These findings indicate that the RepPLRV and CPLMV transgenes do not affect plant productivity.

### 2.6. Assessment of Viral Resistance of Transgenic Potato Lines in a Virus-Endemic Zone

Viral resistance evaluations under natural field exposure were performed in two field trials (2016 and 2017 growing seasons) in Tupungato, Mendoza Province, a potato production region where PVY and PLRV infection rates are normally high. A complete randomized block design with three replicates was implemented in the field to evaluate virus infection in RY21 and RY25 transgenic plants, along with control plants (NT and 2CA2). Progeny tubers harvested from these plants were subsequently sprout-tested for PLRV and PVY infections.

During the first growing season, tubers obtained from the greenhouse were planted. Because climatic conditions were highly unfavorable, tuber production was limited, and most plants generated only a single tuber. A total of 92 tubers produced sprouted plants suitable for serological testing. Initially, ELISA assays were performed, followed by RT-PCR analysis on the same plants. The RT-PCR results corroborated the ELISA findings and, due to their higher sensitivity, were able to detect a greater number of positive plants. In summary, all PVY-positive samples were also found to be infected with PLRV, indicating frequent co-infection. Notably, RT-PCR did not detect any specific amplification products for PVY or PLRV in plants that tested negative for both viruses by ELISA. As shown in Table 1, five out of twenty-one tubers in the NT group tested positive for both PVY and PLRV (23.8%). Similarly, line 2CA2 showed dual infection in four out of eighteen tubers (22.2%), with no statistically significant difference compared to the NT group. In contrast, no infection with either of the evaluated viruses was detected in lines RY21 (0/31) or RY25 (0/22). These differences were statistically significant, indicating that both transgenic lines exhibited complete resistance to PVY and PLRV under field conditions. The overall field infection rates for PVY and PLRV during the 2016 season were approximately 23%, confirming that virus pressure was sufficiently high to reliably assess resistance performance.

During the second trial conducted in Tupungato, tubers obtained during the 2016 season in Malargüe were planted. In the growing season of 2017, weather conditions were considerably more favorable than in the previous year. As a result, all 283 independent plants successfully produced a total of 848 tubers, each of which was evaluated serologically. Remarkably, neither the non-transformed control (NT) nor any of the transgenic lines tested positive for PLRV, indicating that no PLRV infection was detected under field conditions during this season. As shown in Table 2, the incidence of PVY infection was 22.5% in NT controls and 31.5% in the 2CA2 line; this difference was not statistically significant (*p* = 0.2143, Fisher’s Exact Test). In sharp contrast to the susceptible plants, all plants from RY lines (RY21 and RY25) remained completely free of PVY, representing highly significant virus protection compared to NT controls (*p* = 0.0001 and *p* < 0.00001, respectively; Fisher’s Exact Test).

In conclusion, although average infection rates for PVY and PLRV during the 2016 season reached 23.8%, and PVY incidence among susceptible lines reached 31.5% in the 2017 season, the RY transgenic plants consistently demonstrated strong field resistance to both viruses. These results underscore the potential of RY21 and RY25 as valuable candidates for cultivation in virus-endemic regions.

## 3. Discussion

Potato production is significantly affected by viral diseases. More than 50 plant viruses have been reported to infect potatoes [4], with potato virus Y (PVY) and potato leafroll virus (PLRV) being among the most economically damaging, causing substantial yield and quality losses worldwide [4,35,36]. Plant pathologists and breeders have attempted to control viral diseases using various methods to ensure the production of virus-free seed potatoes. These methods include thermotherapy and tissue culture, specific breeding strategies for seed production and storage, seed potato certification programs, and control of viral vectors using insecticides, biopesticides, and mineral oils. However, as these methods are often ineffective and expensive, the development and use of resistant crop cultivars is the most efficient strategy to mitigate the impact of viral diseases in agricultural settings, particularly in developing countries [4,37].

In previous work, we developed transgenic lines of the potato cultivar Kennebec that exhibited resistance to PLRV through the expression of the viral replicase gene (RepPLRV). These lines, including the 2CA2 line, were evaluated through graft inoculation and field trials [30]. However, because multiple viruses frequently co-infect crops in field conditions, it is essential to engineer resistance to more than one virus simultaneously [32,38]. In the present work, to develop stably transgenic potato plants resistant to both PVY and PLRV, transgenic Kennebec plants expressing both the RepPLRV and the capsid protein gene of lettuce mosaic virus (CPLMV), referred to as RY lines, were generated.

First, molecular components potentially involved in the resistance mechanism of the transgenic lines were evaluated. Mapping of sRNAs showed a heterogeneous distribution across the RepPLRV sequence in the RY lines. The high abundance of 21 and 22 nt siRNAs is consistent with PTGS-mediated targeting of this region. On the other hand, given that CP-mediated resistance relies on a direct protein-mediated effect, we analyzed total protein extracts from the RY lines and confirmed high levels of LMV capsid protein expression, with no significant variation among the different transgenic lines. Dinant et al. [27] and Hassairi et al. [29] reported that the presence of CPLMV protein is essential for achieving PVY resistance in transgenic tobacco and potato plants, respectively. In line with these findings, the detection of CPLMV protein in our potato lines supports the hypothesis that the strong resistance to PVY observed in lines RY21 and RY25 is mediated by this protein. Moreover, small RNA sequencing analysis revealed a very low number of sRNAs derived from the CPLMV coding sequence (fewer than 30 reads). This suggests that the observed resistance is unlikely to involve RNA silencing mechanisms directed against the viral coat protein RNA. Mechanical inoculation of potato plants with PVY can lead to systemic infection; however, PLRV requires transmission by aphids or grafting to establish systemic infection. Therefore, under controlled greenhouse conditions, we could only evaluate resistance through direct inoculation with PVY. The transgenic lines RY21 and RY25 showed resistance under high infection pressure from mechanical inoculation, whereas control plants consistently exhibited high levels of PVY infection.

Assessment of tuber yield in the RY transgenic candidate lines under greenhouse conditions, performed over multiple years, demonstrates that expression of the RepPLRV and CPLMV transgenes does not affect plant productivity. Subsequent field trials confirmed that this conclusion could be extended to virus-free field conditions in Malargüe, Mendoza Province. Under field evaluations, the transgenic lines RY21 and RY25 also exhibited agronomic performance comparable to that of the non-transgenic Kennebec cultivar from which they were derived. No significant differences between the transgenic plants and the NT control plants in terms of growth, yield, and tuber shape were observed, indicating that RY lines retain the high levels of productivity and the characteristics of the original commercial cultivar. Moreover, these results were confirmed across three additional consecutive planting campaigns conducted in a field in Buenos Aires Province between 2022 and 2024.

The viral resistance of the transgenic potato lines was further evaluated under natural field exposure in a virus-endemic area. RY21 and RY25 lines, together with their non-transgenic Kennebec control, were cultivated in field trials to assess their resistance to PVY and PLRV, and to compare phenotypic and agronomic performance. These evaluations were conducted over two consecutive growing seasons in Tupungato. During the growing season of 2016, RY transgenic plants exhibited complete resistance to both PLRV and PVY under natural field conditions. In contrast, all susceptible (non-transgenic) plants that tested positive for one virus were also co-infected with the other, resulting in an overall infection rate of 23.81%. Interestingly, in all 2CA2-infected plants, the presence of PLRV and PVY was detected. Although the 2CA2 line was previously reported to be resistant to PLRV [30], the occurrence of infection in this trial may indicate a breakdown of resistance, potentially triggered by co-infection with PVY—a phenomenon previously suggested by Vazquez Rovere et al. [32]. These findings support the central hypothesis of this study: the coexistence of multiple viruses under natural conditions can compromise RNA silencing-based resistance strategies. Even though PLRV resistance was only validated under natural infection in the field during a single growing season, no tubers from the RY21 or RY25 transgenic lines tested positive for PLRV or PVY. Notably, mixed infection with PVY and PLRV can result in yield losses of up to 95%, severely impacting farmer income. Adoption of dual-resistant potato cultivars could restore most of the potential yield, even if the resistance is not complete; assuming 90% effectiveness, income could increase approximately 17-fold compared with severely infected crops.

During the second field trial in Tupungato (2017 growing season), weather conditions were considerably more favorable than in the previous year. PVY infection was detected in 31.5% of the susceptible control plants, whereas all RY transgenic lines remained completely free of PVY, demonstrating a highly significant level of resistance. Unfortunately, PLRV infection was entirely absent from the field. The field trials in Tupungato were conducted near commercial potato fields where insecticides are routinely applied, possibly at higher intensities during the 2017 season. Such treatments may have disrupted PLRV transmission by killing aphids before they could acquire or transmit the virus during the latent period. However, these insecticides are generally ineffective against PVY transmission, as they do not act quickly enough to prevent the rapid virus transfer by transient aphids [39]. This situation could therefore explain why PVY, but not PLRV, was detected in susceptible lines during the 2017 field evaluation. In the future, it would be very interesting to determine how the yield of non-transgenic (NT) varieties is affected in comparison to transgenic varieties in a mixed secondary infection, where the more relevant yield losses are expected to occur. In both growing seasons in Tupungato, we were unable to record significant differences in yields, most likely due to the climatic conditions of 2016, the stage of plant growth at the time of infection, and the absence of PLRV in the second season. It is well known that planting infected tubers is historically the cause of “the greatest yield loss in food crops” before the widespread adoption of seed certification. 

Kennebec potatoes are a popular and important potato variety due to their versatile culinary uses, fungal disease resistance, and high yield. Although numerous authors obtained immunity against PVY by transgenesis [29,40,41,42], to our knowledge, this is the first report describing the development of Kennebec transgenic lines highly resistant to PVY. Moreover, in this study, the 2016 field trial demonstrated that the transgenic lines RY21 and RY25 exhibited immunity to PLRV. Remarkably, this resistance was stably maintained after several years of in vitro propagation and through at least three tuber generations. To date, PLRV immunity maintained across clonal generations of *S. tuberosum* has only been reported by Orbegozo et al. [18], who generated transgenic ‘Desiree’ plants expressing a hairpin construct derived from the PLRV coat protein gene. However, these plants were not evaluated in field conditions, where multiple viruses frequently co-infect crops, and this resistance strategy may be susceptible to breakdown, as indicated by our observations of the 2CA2 line in the present study. Compared with current CRISPR-based resistance strategies, the dual-transgene approach described in this study offers distinct and complementary advantages. Although CRISPR/Cas systems have been employed to engineer virus resistance by targeting viral genomes or host susceptibility genes, their application can be constrained by delivery challenges, off-target effects, regulatory uncertainty, and, most critically, the potential emergence of escape mutants [43,44]. In contrast, the dual-transgene strategy relies on the stable expression of well-characterized resistance genes, reducing the likelihood of resistance breakdown and limiting viral escape through mutation or host repair mechanisms. Its strong field validation and greater regulatory familiarity further support its continued relevance as a robust and complementary option as CRISPR technologies mature.

It is worth noting that achieving durable resistance to PLRV under field conditions has historically been challenging. In 1988, Kaniewski and Thomas [45] reported that CP-expressing Russet Burbank plants were the first to exhibit resistance to PLRV; however, the only line that remained un-infected in growth chamber assays became completely infected when exposed to natural field conditions [46]. This highlights the importance of field evaluations to assess the stability of virus resistance and agronomic performance under natural conditions, which can differ substantially from greenhouse outcomes and are essential for pre-commercial biosafety assessments. Transgenic crops engineered for resistance raise biosafety concerns, particularly regarding the potential transfer of transgenes to wild potato relatives through pollen. Such gene flow may lead to unintended ecological consequences, including the emergence of “superweeds” or disruptions to natural populations. However, a field study conducted in Argentina investigating cross-pollination between cultivated potatoes and wild *Solanum* species found no evidence of transgene transfer under natural conditions [42]. Beyond this experimental evidence, it is well established that, in tetraploid crops such as *S. tuberosum*, hybridization with wild diploid relatives typically results in sterility. Furthermore, the potato is, in practical terms, a clonal crop, since multiplication practices of potato seeds are performed by tubers and not by true seeds. Another concern is that transgenic virus-resistant potatoes may exert strong selection pressure on viral populations, potentially accelerating the emergence of resistance-breaking variants. However, evidence suggests that strategically targeting essential viral genes can significantly slow this process [47], supporting an optimistic outlook for the durability of our transgenic lines. Furthermore, these transgenic lines are well-suited to integration into Integrated Pest Management (IPM) programs, as they reduce dependence on chemical pesticides while complementing existing cultural and chemical control practices. In addition, they may enhance seed certification systems by enabling the production of pathogen-free planting material, although their implementation requires robust and well-defined regulatory frameworks.

Transgenic potato plants simultaneously resistant to PLRV and PVY have been previously obtained, and different levels of tolerance to these viruses have been reported only in greenhouse conditions [38,48]. The lack of validation in the field raises questions about the actual performance of viral resistance under natural conditions where viral pressure, environmental variability, and vector dynamics differ substantially from controlled settings. In the present work, data obtained in the experimental field of Tupungato, a virus-endemic zone, showed that total resistance against PLRV and PVY was achieved in transgenic RY lines of *S. tuberosum* cv. Kennebec. These findings demonstrate the durability and robustness of this biotechnological strategy, confirming its ability to provide dual viral resistance in real agricultural environments.

## 4. Materials and Methods

Construction of LMV-REP plasmid

For the construction of the binary vector pZP-Kana-CPLMV-RepPLRV, the plasmid pBS-LMV (provided by INRA Versailles), containing the sequence coding for LMV capsid under the control of the Cauliflower Mosaic virus 35S (CaMV35S) promoter and the nopaline synthase gene terminator (Tnos), was modified by adding the CaMV 2×35S promoter and the rubisco gene terminator (2x35S-Trbs) cassette from the plasmid pKYLX. The complete PLRV-ORF2 sequence from the Argentinian PLRV isolate (PLRV-Ar) (referred to as RepPLRV, GenBank accession AF220151.1) was inserted into the XhoI restriction site of pBS-LMV- pKYLX vector between the 2X35S and the 2x35S-Trbs. The CPLMV-RepPLRV cassette obtained was released by restriction with the enzymes SpeI and NruI and subcloned into Xbal and HindIII sites of the pZP-Kan binary vector, which carries the nptII gene that allows the selection of plants in media supplemented with kanamycin. The final construct, named pZP-Kan-CPLMV-RepPLRV, was verified by restriction enzyme digestion and was transferred into *Agrobacterium tumefaciens* LBA4404 (pAL4404) strain following the protocol of electroporation [49].

Potato cv. Kennebec transformation and regeneration

Leaf discs of *Solanum tuberosum* cv. Kennebec were co-cultured with the *A. tumefaciens* LBA4404 pAL4404 carrying the pZP-Kan-CPLMV-RepPLRV construct, as previously described by Vazquez Rovere et al. [30]. Transgenic RY plants, as well as 2CA2 line [30] were maintained in vitro by periodic micropropagation in growing chambers (CMP 3244; Conviron, Winnipeg, MB, Canada) at 18–22 °C, under 8/16 h dark/light cycle. Plants were subsequently transferred to soil, in 8-liter pots, and grown under greenhouse conditions for evaluation of growth parameters: virus testing and/or tuber production. Tubers were harvested and kept in darkness at 4 °C for up to 6 months before sown in field trials or used directly for the different analyses performed in this study.

Plant DNA and RNA extraction, cDNA synthesis, and RT-PCR

Genomic DNA from kanamycin-resistant plants was extracted according to Dellaporta et al. [50]. PCR was performed to confirm the presence of the RepPLRV, using primers 5′RepcATG (ATGGGATTACGGTCTGGAGA) and 3′Rep2 (TCAGTGTTCTTTTGTGGTGGCACTCGGA), and the CPLMV, using CP Up (ACTCTAGAGGATCCAAGCTTTATTTTTACAACAATTACCAACAACAAC) and CP low (TTAGTGCAACCCTCTCACGCCTAAGAGAGTATGCATATTCTGATTTACATC) primers. RNA from leaf tissue was isolated using the Trizol commercial extraction system (Invitrogen, Carlsbad, CA, USA). For cDNA synthesis, 1 μg of RNA was treated with DNase I (Thermo Fisher Scientific, Norristown, PA, USA) and cDNA was synthesized using random primers and SuperScript III reverse transcriptase (Thermo Fisher Scientific) according to the manufacturer’s instructions. cDNAs were amplified by PCR with specific primers: 5′RepcATG and 3′Rep2 for RepPLRV and CP Up and CP low for CPLMV. Amplification of RepPLRV genomic DNA or cDNA was performed under the following conditions: a denaturation step at 95 °C for 1 min, followed by 35 cycles of 1 min at 94 °C, 1 min at 55 °C, and 2 min at 72 °C with a final extension at 72 °C for 10 min. CPLMV from genomic DNA or cDNA was amplified as follows: a denaturation step at 95 °C for 1 min, followed by 35 cycles of 1 min at 94 °C, 1 min at 55 °C, and 1 min at 72 °C with a final extension at 72 °C for 10 min. RNA from the 2CA2 line [30] was used as a positive control.

Plant total protein extraction and Western blot

Two-week-old in vitro-grown plants were transplanted into 5 L pots with soil and acclimatized for 2–3 weeks. Potato leaf samples were ground in mortars with liquid nitrogen until a fine powder was obtained. Total plant proteins were extracted using buffer 100 mM KCl, 5 mM MgCl_2_, 400 mM Sucrose, 100 mM TrisHCl pH 8, 10% glycerol, 10 mM β-Mercaptoethanol, and 2 mM PMSF) [51], at a ratio of 300 mL per 100 mg of tissue. The mixture was incubated on ice for 10 to 30 min and centrifuged at 4 °C for 20 min at 12,000 rpm. The supernatant was transferred to a clean tube and stored at −80 °C until use. Proteins were separated on 12% SDS-PAGE before blotting on Hybond ECL nitrocellulose membrane (GE Healthcare, Beijing, China). CPLMV protein was detected using an anti-CP rabbit monoclonal primary antibody (Bioreba, Reinach, The Netherlands), followed by an alkaline phosphatase-conjugated anti-rabbit secondary antibody (Merck, Rahway, NJ, USA), and bands were visualized using NBT/BCIP reagents (Promega, Madison, WI, USA).

Detection of viral RNA

Two-week-old in vitro-grown plants were transplanted into 5 L pots with soil and acclimatized for 2–3 weeks. Total RNA was extracted from potato leaf tissue as described above. PCR assays for PLRV detection were performed with Platinum Taq DNA Polymerase (Invitrogen) and specific primers: PLpP0up (ATGATTGTATTGACCCAGTC) and PLpP0low (TCATTCTTGTAATTCCTTTTGGAG), which amplify the complete ORF 0 sequence, or PLF (ACDGAYTGYTCYGGTTTYGACTGG) and PLR (TCTGAWARASWCGGCCCGAASGTGA), which amplify the intergenic region of the virus. The reactions were carried out with a denaturation step at 94 ◦C for 4 min, followed by 40 cycles of 1 min at 94 °C, 1 min at 55 °C, and 1 min at 72 °C, with a final extension at 72 °C for 10 min. PCR assays for PVY RNA detection were performed as described above, using the primers Hc-Pro up (GCGGCAGAAACACTCGTCG) and HC-Pro low (CCTGGGCGCTTCGGCCCAAG). In all cases, amplified products were purified using a QIAEX II Gel Extraction Kit (Qiagen, Venlo, The Netherlands), cloned into pGEM^®^-T Easy Vector (Life Technologies, Carlsbad, CA, USA), and sequencing service was performed at the National Institute of Agricultural Technology, Biotechnology Institute, Genomics Unit.

Small RNA sequencing and bioinformatic analysis

Two-week-old in vitro-grown plants were transplanted into 5 L pots with soil and acclimatized for 6 weeks. Small RNA libraries were prepared from total RNA using the Qiagen QIAseq miRNA Library Prep Kit and sequenced on an Illumina NovaSeq 6000 (paired-end PE75 × 50) (High-Throughput Genomics Shared Resource at the Huntsman Cancer Institute, University of Utah). Sequencing service was performed at the National Institute of Agricultural Technology, Biotechnology Institute, Genomics Unit. Only Read 1 was used for downstream sRNA analyses. Adapter trimming and quality/length filtering were performed with cutadapt (3′ adapter: AACTGTAGGCACCATCAAT; -q 30 -m 18 -M 26), retaining reads in the 18–26 nt size range. Libraries yielded 51.6–56.1 million raw R1 reads per sample, and 43.8–46.2 million reads per sample after trimming/size selection. Filtered reads were mapped to the RepPLRV (ORF2b; ~1.85 kb) reference (and, when indicated, to the complete PLRV genome, GenBank X74789.1) using ShortStack (Bowtie backend) with zero mismatches (--mismatches 0) and unique placement of multi-mapping reads (--mmap u). BAM files were sorted and indexed with SAMtools, and coverage/positional patterns were visualized with sRNA_Viewer (https://github.com/MikeAxtell/sRNA_Viewer, accessed on 13 June 2023). NCBI BioSample accessions are: SAMN54431893 (NT), SAMN54431894 (RY21), and SAMN54431895 (RY25).

Plant inoculation and assessment of virus resistance in greenhouse

PVY infection trials were conducted under biosafety greenhouse conditions. Two-week-old in vitro-grown plants were transplanted into 5 L pots with soil and acclimatized for 3 weeks. The N used was variable for non-infected (NI) plants (4 NT and 8 for transgenic lines) and for infected (I) plants (10 NT and 16 for transgenic lines). NT and 2CA2 plants were used as controls.

Plants were mechanically inoculated, scraping the epidermal leaf layer with Carborundum and applying a mixture of field PVY-infected potato foliar extract, collected in Tupungato (2007, 2008, 2016, and 2017). Inocula were confirmed as PVY-positive by ELISA and RT-PCR. Subsequently, leaves were washed with water to remove excess abrasive and inoculum. After a week, inoculation was repeated to ensure PVY infection.

Inoculated and uninoculated (used as healthy controls) plants were maintained under a 16 h day length, and day/night temperatures of 24 ± 2 °C. Symptoms were monitored throughout the experiment, and PVY infection was assessed three weeks post-inoculation using a commercial DAS–ELISA test according to the manufacturer’s instructions (Bioreba). Negative and positive controls of the kit were employed. Assays were conducted three times and analyzed separately. Uninoculated plants were used as healthy controls. Spectrophotometric readings at 405 nm were performed with Multiskan Spectrum equipment (Thermo Fisher Scientific), with air blanking, after different times of substrate reaction, until the highest values were about A405 =1.5–2.0 (between 0.5 and 2 h. of reaction). Twice the mean of the absorbance values of the healthy control was chosen to consider positive for PVY infection.

Field trials evaluations

Candidate resistant transgenic plants were subjected to field trials to analyze agronomic performance and genetic stability of resistance to PLRV and PVY. For each field trial, prior to sowing, the soil was cleared, and the furrows were prepared. Virus-free tubers were stored at low temperature for at least 60 days after harvest. A complete randomized block design with three replicates was used to evaluate the transgenic lines and NT controls. Tubers were planted in rows 4 m long, spaced every 2 m, at a depth of 2 or 3 cm below the soil surface. A total of 24 m^2^ was planted for each of the transgenic lines or with non-transgenic (NT cv. Kennebec or Spunta) tubers. Average fresh tuber weight obtained (in grams (g)) calculated from the mean value (total tuber weight divided by total number of plants/tubers) of each block. The plots were fenced to avoid vandalism and to prevent access by large animals. Comparative field trials to evaluate the agronomical performance of RY lines were carried out during the growing seasons of 2005–2006 and 2015–2016 at Malargüe, Mendoza Province, which is a virus-free potato seed production region of Argentina. Three additional consecutive planting campaigns were carried out in a field in Buenos Aires Province, Argentina, between 2022 and 2024. Viral resistance evaluations under natural field exposure were performed in Tupungato, Mendoza Province (Argentina), which is a potato production region in which PVY and PLRV infection rates are normally high. These trials were carried out during two consecutive growing seasons (2015/2016 and 2016/2017). Regarding the PLRV strains circulating at the endemic site during that period, the detected isolate corresponded to the Argentinian strain of PLRV (PLRV-Ar; GenBank accession number KY856831). Furthermore, the PVY strain present in the Mendoza experimental field coincided with the one that had been previously identified by Barrios Barón as a recombinant NTN type isolate (recombinant PVYNTN) [34]. For all trials, the number of plants grown in the field was recorded for each replicate block. Both trials were harvested manually in the autumn, collecting tubers from individual plants separately to avoid mixing. Tubers harvested from each replicate block were bulked by line, then counted and weighed. Tubers were stored at 4 °C until their evaluation. Viral diagnosis was performed by ELISA tests by the Potato Seed Analysis Laboratory of INTA Balcarce. Harvested tubers were sprout-tested. For this test individual buds were excised from tubers and planted in a greenhouse. Emerged young plants were assessed for virus infection by recording symptoms and by ELISA at 2–4 weeks after emergence. In selected cases, results were validated using RT-PCR. Agronomic performance of transgenic lines was compared to that of NT Kennebec and Spunta controls. Biological containment of field trials, assay isolation, and disposal of transgenic materials were performed as established by the guidelines of the National Commission on Agrobiotechnology (CONABIA, Argentina, S01:0053198/2005; S01:045507/07; S05:0058167/2014; Expte:2020-72180378).

Statistical analysis

Two-way ANOVA was used for ELISA test statistical analysis using Graphpad Prism 8 software (USA). Statistical significance was set at a value of *p* < 0.05. The agronomic performance was analyzed using the nonparametric Kruskal–Wallis test implemented in InfoStat (http://www.infostat.com.ar, accessed on 19 November 2025). Statistical significance was set at a value of *p* < 0.05. Fisher’s exact test was used for comparing each line against NT in the viral resistance evaluation, where (*) denotes *p* < 0.05.

## 5. Conclusions

Our results demonstrate that transforming Kennebec potato plants with a construct co-expressing the full, unmodified coding sequences of PLRV ORF2 and the LMV capsid, each providing a distinct resistance mechanism, provides effective and stable resistance to two of the most harmful potato viruses, PLRV and PVY, across multiple seasons and locations. This strategy represents a significant advance in potato biotechnology, offering an environmentally friendly alternative to chemical vector control while preserving the integrity and agronomic performance of the commercial cultivar under both high-pressure controlled conditions and natural infection pressures.

## Figures and Tables

**Table 1:** Evaluation of PLRV and PVY incidence during the 2016 field trial in Tupungato by ELISA and RT-PCR.

Line	NT	2CA2	RY21	RY25
Plants (tubers)	21	18	31	22
PLRV-positive tubers	5	4	0	0
PVY-positive tubers	5	4	0	0
PLRV and PVY incidence (%)	23.8	22.2	0	0
Fisher’s Exact Test (vs. NT)	—	1	0.0078 *	0.0211 *

(*) denotes *p* < 0.05. NT, non-transgenic; 2CA2, transgenic line for RepPLRV; RY21 and RY25, transgenic lines carrying both RepPLRV and CPLMV.

**Table 2:** Serological evaluation of PLRV and PVY incidence during the 2017 field trial in Tupungato.

Line	NT	2CA2	RY21	RY25
Plants (tubers)	89 (275)	73 (176)	49 (148)	72 (249)
PLRV-positive tubers	0/275	0/176	0/148	0/249
PVY-positive tubers	36/275	26/176	0/148	0/249
PVY incidence (%)	22.5	31.5	0	0
Fisher’s Exact Test (vs. NT)	—	0.2143	0.0001 *	<0.00001 *

(*) denotes *p* < 0.05. NT, non-transgenic; 2CA2, transgenic line for RepPLRV; RY21 and RY25, transgenic lines carrying both RepPLRV and CPLMV.

## Data Availability

The original contributions presented in this study are included in the article. Further inquiries can be directed to the corresponding authors. All the data are publicly available. Germplasm accessions are available at INTA’s active germplasm banks of IABIMO, INTA Castelar.

## Abbreviations

The following abbreviations are used in this manuscript:

PVY	potato virus Y
PLRV	potato leafroll virus
CPLMV	lettuce mosaic virus capsid protein
TMV	tobacco mosaic virus
PVX	potato virus X
CMV	cucumber mosaic virus
TRV	tobacco rattle virus
NT	non-transgenic control plants
*nptII*	neomycin phosphotransferase II
CRISPR	Clustered Regularly Interspaced Short Palindromic Repeats
RT-PCR	reverse transcriptase polymerase chain reaction
siRNA	small interfering RNA
NGS	next-generation sequencing
ELISA	enzyme-linked immunosorbent assay
OD	optical density
